# Design and Control of Multi-Plate MR Clutch Featuring Friction and Magnetic Field Control Modes

**DOI:** 10.3390/s22051757

**Published:** 2022-02-23

**Authors:** Jin-Young Park, Jong-Seok Oh, Young-Choon Kim

**Affiliations:** 1 Department of Mechanical Engineering, Kongju National University, Cheonan 31080, Korea; pjy0186@kongju.ac.kr; 2 Department of Future Automotive Engineering, Kongju National University, Cheonan 31080, Korea

**Keywords:** magnetorheological (MR) fluid, multi-plate MR clutch, torque transmission, magnetic field control mode

## Abstract

A magnetorheological (MR) multi-plate clutch was proposed with both mechanical friction mode and magnetic field control modes. The magnetic field control mode was based on an MR fluid coupler that changed its viscous properties according to the density of an applied magnetic field. This mode was used in the early stage of clutch operation to reduce the impact of friction between the disc and plate, and eliminate to the extent possible the difference in their relative speeds when contacting each other in later stages. Once the rotational speed difference between the disc and plate was reduced, the clutch was operated in mechanical friction mode by compressing the friction surfaces together. A torque modeling equation was then derived for each mode based on the Bingham model of the MR fluid, and the transmission torque of the proposed multi-plate clutch was derived using these equations as well as magnetic field analysis results obtained using ANSYS Maxwell. A multi-plate MR clutch was then fabricated, and its torque transmission characteristics were evaluated in the magnetic field control and mechanical friction modes. The results confirmed that the model-based torque calculations were consistent with the observed transmission torque. Finally, control algorithms for mechanical friction only and mixed mechanical friction/magnetic field control torque tracking of the proposed MR multi-plate clutch were designed, and their performances were evaluated when applying unit step command, half-sine-wave command, and rotational speed changes. The results indicated that the torque tracking control was performed smoothly, demonstrating the advantages of the proposed clutch.

## 1. Introduction

Interest in high-performance power transmission components, such as clutches, has recently been increasing owing to the demands for high-quality power transmission systems. Most power transmission components use multiplate friction clutches to provide high power and smooth transmission characteristics; however, noise and vibration are generated by the friction between the clutch disc and plate, potentially decreasing rider comfort. Research and development of new friction clutch systems is therefore actively underway to address this shortcoming, and has to date primarily focused on improving the control methods and reducing friction noise and vibration using algorithms. For example, Vasca et al. proposed a torque transmission model for a dry clutch that applied the effect of clutch spring characteristics [1]; Jibin et al. analyzed the effects of contact angle and drag torque according to the friction plate pattern and wet clutch spacing [2]; Agostino et al. applied various slip control methods to realize the soft combination of dry clutch and power [3]; and Desmidt et al. analyzed a power transmission system to propose a weight optimization method for a multiplate clutch in a two-speed rotorcraft transmission [4]. As demonstrated by these examples, many studies have attempted to realize smooth clutch fastening, but it remains impossible to ideally reduce the shock and vibration that occur during the clutch fastening process. New control methods and materials to address this issue are therefore the subject of intense ongoing research.

One such material, magnetorheological (MR) fluid increases its resistance to flow under a magnetic field load. As the yield stress of the MR fluid changes according to the applied magnetic field, efforts have been undertaken to apply it in a wide variety of systems, including dampers, valves, mounts, clutches, and brakes. Two MR fluid modes have been widely used to date according to the fluid stimulation mechanism and relative motion. The first is the flow mode, in which the fluid moves through fixed stimuli (such as in a damper, valve, or mount); in this mode, changes in the pressure and flow rate of the fluid in a pipe occur simultaneously. The second is the shear mode or rotational shear mode, in which the fluid passes between two parallel plates, one of which is fixed and the other of which rotates or moves (such as in clutch or brake systems). Notably, Mukhopadhyay et al. investigated the effect of plate orientation on MR fluid behavior in shear mode under a magnetic field [5].

Ongoing research on MR fluids has improved their performance to the extent that they have been successfully applied in various clutches. Neelakantan and Washington proposed a method applying the MR sponge concept to solve problems related to the centrifuging of the MR fluid owing to clutch rotation [6]. Benetti and Dragoni performed design and electromagnetic field analyses to develop a 100 Nm-class multiplate MR brake [7]. Gratzer et al. suggested an efficient MR clutch that combined the clutch design for an all-wheel drive vehicle with fluid development and magnetic circuit optimization [8]. Kikuchi et al. suggested a design method for an MR multiplate clutch with a 50 μm MR fluid layer between the input and output plates, and presented the results of an experimental evaluation [9]. Zhang et al. proposed a design method using nondimensional parameters for a large-capacity MR single-plate clutch [10]. Güth et al. suggested a new design concept combining a variable-speed electric actuator and MR clutch [11]. Törőcsik studied the transmission torque and corresponding power consumption according to the number and thickness of plates used in an MR multiplate clutch [12]. Wang et al. proposed a mechanical method for increasing the number of plates to generate a high transmission torque with an MR multiplate clutch [13]. Kavlicoglu et al. analyzed the output torque of a single-plate clutch employing an MR grease with six different iron particle sizes [14]; they also developed a double-plate prototype of an MR clutch after conducting a performance design using a variable torque range according to changes in input current [15]. Thakur and Sarkar proposed a simulation-based method to estimate the maximum transmission torque and response time of an MR clutch using the shear mode of the MR fluid [16]. Yadmellat et al. presented simulation and experimental results describing the performance of an MR clutch using the Preisach model, constituting a hysteresis model approach [17]. Bucchi et al. suggested a method for improving torque characteristics by applying a neural network to an MR clutch controller [18]. Pilch proposed an MR fluid for an optimal clutch design based on simulation results to realize variable controllability according to temperature [19]. Leong et al. proposed a method for the design of a magnetic coupler to amplify axial speed using magnetic attraction [20]. Fernando suggested the use of magnetic field modulation and an EM clutch slip controller through d–q current control [21]. Kavlicoglu et al. proposed a design and slip controller for a multiplate limited-slip differential clutch to achieve high torque transmission using an MR fluid [22]. Rizzo et al. proposed a method to improve the transmission torque of an MR fluid clutch using the eddy current induced in the conduction region by relative rotation [23]. Latha et al. proposed MR fluid coupler type multiplate clutch to overcome mechanical friction [24]. Olszak et al. designed a hydrodynamic clutch that incorporated an MR fluid and presented the results of simulations and experiments considering friction loss [25]. Zhang et al. designed the magnetic field structure of a new dual clutch and suggested a relationship between torque and current using a finite element analysis [26]. Finally, Kluszczynski and Pilch compared the clutch torque per total volume from the perspectives of torque, diameter, external length, and ratio according to the number of plates in an MR clutch [27].

Recently, Park et al. developed an MR fluid-based multiplate clutch that provided the advantages of both magnetic field control mode and mechanical friction mode, a notable advance beyond the conventional MR multiplate clutch, which only supports magnetic field control mode [28]. An optimal design of this clutch was previously achieved using torque modeling of mechanical friction mode and an electromagnetic field analysis of magnetic field control mode, and its performance was evaluated in each mode. However, torque tracking control employing both the magnetic field control and mechanical friction modes has yet to be evaluated for the proposed clutch.

Indeed, the following outstanding research issues related to MR clutches were identified by the authors:

1. There is no existing MR clutch capable of transmitting power by compression force;
2. There is a lack of research on hybrid MR clutches that employ two operating modes;
3. The MR fluid clutch can transmit high power and provide smooth engagement but requires a large size or high current; however, studies on the minimum size or current have been lacking;
4. Existing studies have thus far failed to verify the torque transmission and control performance of the MR fluid clutch in both magnetic field control and mechanical friction modes.

Therefore, in this study, the rotational shear mode—a characteristic of the MR fluid—was used to realize the magnetic field control of a multi-plate MR clutch, while the operation mode of a conventional friction clutch was used to realize the mechanical friction mode. To do so, torque modeling and electromagnetic field analysis of the MR single-plate clutch were performed, and the transmission torque was identified by fabricating a prototype multi-plate clutch. The applied current and compression forces were then controlled to investigate its torque tracking performance using an algorithm designed to realize smooth multi-plate MR clutch operation.

## 2. Torque Modeling of the Multi-Plate MR Clutch

**Figure 1:**
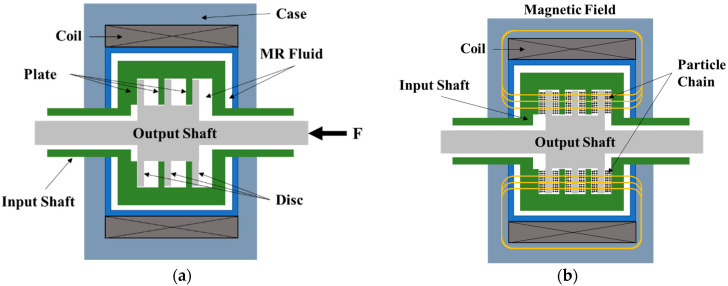
Operational concept of the multi-plate MR clutch: (**a**) mechanical friction mode; (**b**) magnetic field control mode.

The MR multi-plate clutch proposed in this study was able to operate in one of two modes—mechanical friction or magnetic field control—by moving the output shaft in the axial direction, as shown in Figure 1. In mechanical friction mode, shown in Figure 1a, power is transmitted from the input shaft to the output shaft through the friction between the input and output plates, similar to the operation of a conventional general clutch. In magnetic field control mode, shown in Figure 1b, power is transmitted between the input and output plates through the shear stress induced in the MR fluid by sliding in its shear mode according to its properties as dictated by the applied magnetic field. In this section, the properties of the MR fluid are first briefly discussed, then the torque modeling in each mode is described based on these properties.

### 2.1. Modeling of MR Fluid

Generally, MR fluids consist of magnetic (typically iron) particles suspended in a low-permeability solvent carrier fluid; the particles used in this study were micron-sized iron particles. The resistance of the MR fluid to flow increases under an applied magnetic field load. When there is no magnetic field load, an MR fluid exhibits the same behavior as a Newtonian fluid, in which the suspended particles move freely. However, in the presence of a magnetic field load, an MR fluid exhibits Bingham fluid behavior, presenting a yield strength as the particles are charged to form a chain structure within the fluid. Thus, the rheological properties of an MR fluid change with the magnetic field strength. The shear stress in the MR fluid *τ* can therefore be predicted using the Bingham model as follows [29,30]:

(1) $$\tau =\tau_{y}+\eta \dot{\gamma }$$

where $\tau_{y}$ and η denote the yield stress and viscosity coefficient after MR fluid yielding, respectively, and $\dot{\gamma }$ is the shear rate of the MR fluid at its interface with the discs and plates. Because the gap between each torque input plate and torque output disc is very small, a linear distribution of shear can be assumed. Thus, the shear rate of the MR fluid at its interfaces can be determined as follows:

(2) $$\dot{\gamma }=\frac{r\omega }{d}$$

where r and d are the radius of the smallest area on the disc and the gap between the disc and plate, respectively, and ω is the difference in angular velocity between the disc and plate. The yield shear stress in the MR fluid represents the shear stress at the moment the fluid begins to flow when under an applied magnetic field. Because the fluid flow begins in a very short time and exhibits complex behaviors, it is difficult to accurately measure the corresponding yield shear stress. Thus, a linear equation for shear rate and shear stress was derived, and the shear stress when the shear rate was zero was defined as the dynamic yield stress. In general, the dynamic yield stress can be treated as the yield shear stress of the MR fluid and is expressed as a function of the magnetic field intensity.

Previous studies have ignored the claim that the viscosity of the MR fluid has a significant effect on the applied magnetic field. To consider this issue in the present study, the approximate constant was used as a unique characteristic value determined by the type of solvent and particles in the MR fluid, their composition ratio, and the composition environment [31,32] as follows:

(3) $$\eta =\eta_{\infty }+(\eta_{0}-\eta_{\infty })(2e^{-B\sigma_{S\eta }}-e^{-2B\sigma_{S\eta }})$$

(4) $$\tau_{y}=\tau_{y\infty }+(\tau_{y0}-\tau_{y\infty })(2e^{-B\sigma_{S\tau_{y}}}-e^{-2B\sigma_{S\tau_{y}}})$$

where $\eta_{\infty }$ and $\eta_{0}$ respectively denote the saturated and natural states of the viscosity coefficient, $\tau_{y\infty }$ and $\tau_{y0}$ respectively denote the saturated and natural states of the yield stress, $\sigma_{S\eta }$ is the saturated moment for the viscosity coefficient, $\sigma_{S\tau_{y}}$ is the saturated moment for the yield stress, and B is the magnetic field density applied to the MR fluid.

Compared to Ferrofluids [33], MR fluids consist of magnetic (typically iron) particles in a carrier fluid. In the presence of a magnetic field, the micron-sized particles link and change the fluid to a semi-solid in milliseconds. When the magnetic field is removed, the fluid just as quickly reverts back to its natural free-flowing state.

The unique characteristics of the MRF-132DG MR fluid (Lord Cop.) used in this study are listed in Table 1. For more detailed information, please refer to technical data from Lord Corp. [34].

### 2.2. Torque Modeling of Mechanical Friction Mode

A multiplate clutch has N friction surfaces, and its size and weight are different from those of a single-plate clutch. However, torque modeling of a single-plate clutch was performed in this study to design the proposed multi-plate MR clutch, assuming that the size and weight were the same to simplify the mathematics. It was assumed that the single-plate clutch is composed of input and output shafts, with a friction pad in between.

The transmission torque of the clutch can be obtained through torque modeling, and the axial compression force *F* applied to the cross section of the clutch plate can be expressed as follows:

(5) $$F=\int_{r_{i}}^{r_{o}}2\pi rpdr=\pi p(r_{o}^{2}-r_{i}^{2})$$

where p is the pressure acting on the friction pad cross section when the clutch is connected, assumed to act uniformly per unit area, and $r_{i}$ and $r_{o}$ are the inner and outer diameters of the clutch friction pad, respectively.

The transmission torque $T_{M}$ generated by the compression between clutch disc and plate in a single-plate clutch is described as follows:

(6) $$T_{M}=\int \int_{r_{i}}^{r_{o}}r\mu \mathrm{sgn}(\omega )dFdr=\frac{2\pi \mu p}{3}(r_{o}^{3}-r_{i}^{3})\mathrm{sgn}(\omega )=\frac{3F\mu (r_{o}^{3}-r_{i}^{3})}{3(r_{o}^{2}-r_{i}^{2})}\mathrm{sgn}(\omega )$$

where μ is the friction coefficient of the clutch friction pad, defined as a function of the plate’s angular speed ω and pressure p. For a more detailed explanation of torque modeling of mechanical friction mode, please refer to the authors’ previous study [28].

### 2.3. Torque Modeling of Magnetic Field Control Mode

In magnetic field control mode, the torque generated by the magnetic field induced viscosity of the MR fluid is transmitted from the friction surface of the input shaft to the friction surface of the output shaft. The transmitted torque is generated by the friction of the MRF acting on its interfaces with the disc and plate as well as the outer annular enclosure. The magnetic field control mode torque $T_{MR}$ of an MR single-plate clutch can be determined by the yield shear stress $\tau_{y}$ and friction area of the MR fluid and can therefore be derived from the properties of the MR fluid as follows:

(7) $$T_{MR}=\int_{A}\tau rdA=2\pi \int_{r_{i}}^{r_{o}}\tau r^{2}dr$$

Substituting Equations (1) and (2) into Equation (7) and rearranging, the following equation can be obtained:

(8) $$T_{MR}=2\pi \int_{r_{i}}^{r_{o}}\tau_{y}r^{2}dr+2\pi \int_{r_{i}}^{r_{o}}r^{2}\eta \left(\frac{r\omega }{d}\right)dr$$

Shear stress is also generated in the MR fluid in the annular enclosure between the ends of the disc and plate of the multi-plate MR clutch. Hence, similar to the MRF fluid in the disc–plate gap, using the average magnetic density obtained from numerical integration and assuming a linear distribution of the shear rate in the gap, the following expression can be obtained [32]:

(9) $$T_{MR}=2\pi r^{2}l\left(\tau_{y}+\eta \frac{r\omega }{d}\right)$$

where r and l denote the radius and length of the annular enclosure, respectively. 

When no magnetic field is applied to the MR fluid, the torque transmission equation of the MR single-plate clutch is given as follows [35,36,37]:

(10) $$T_{0}=\frac{\pi \eta_{0}}{h}(r_{o}^{4}-r_{i}^{4})\omega +\frac{4\pi \tau_{y0}}{3}(r_{o}^{3}-r_{i}^{3})+2\pi r_{o}^{2}t_{d}(\tau_{y0}+\eta_{0}\frac{r_{o}\omega }{h})$$

The torque Equations (6)–(10) for a single-plate clutch were used to determine the torque model of the multiplate clutch designed in this study. Because the multi-plate MR clutch has *N* friction surfaces, the mechanical friction mode transmission torque $T_{M\_m}$ of the multiplate clutch can be expressed as:

(11) $$\begin{array}{l} T_{M\_m}=T_{M}+T_{0}=\frac{3F\mu (r_{o}^{3}-r_{i}^{3})}{3(r_{o}^{2}-r_{i}^{2})}\mathrm{sgn}(\omega )N\\ +\sum_{I=0}^{n}\frac{\pi \eta_{0I}}{h}(r_{o}^{4}-r_{i}^{4})\omega +\frac{4\pi \tau_{y0I}}{3}(r_{o}^{3}-r_{i}^{3})+2\pi r_{o}^{2}t_{d}\left(\tau_{y0I}+\eta_{0I}\frac{r_{o}\omega }{h}\right) \end{array}$$

where h is the gap between the disc and plate, $t_{d}$ is the thickness of the disc and plate, $\tau_{y0I}$ and $\eta_{0I}$ are the *i*th values of $\tau_{y0}$ and $\eta_{0}$, respectively, and *n* is the total number of gaps between discs and plates.

However, because torque transmission between the disc and plate is performed by the MR fluid according to the applied magnetic field, the torque must be calculated at every location in the fluid. Thus, the magnetic field control mode transmission torque $T_{MR\_m}$ is expressed as the sum of all torques acting in the gap between the disc and plate and can be expressed as:

(12) $$\begin{array}{l} T_{MR\_m}=\sum_{I=0}^{n}\left\{T_{MR}+T_{th}+T_{0}\right\}\\ =\sum_{I=0}^{n}\left\{\begin{array}{l} \frac{2\pi \tau_{yI}}{3}(r_{o}^{3}-r_{i}^{3})+\frac{\pi \eta_{I}\left(\frac{\omega }{d}\right)}{2}(r_{o}^{4}-r_{i}^{4})\\ +2\pi r_{o}^{2}t_{d}\left(\tau_{yI}+\mu_{I}\frac{r_{o}\omega }{h}\right)\\ +\frac{\pi \eta_{0I}}{h}(r_{o}^{4}-r_{i}^{4})\omega +\frac{4\pi \tau_{y0I}}{3}(r_{o}^{3}-r_{i}^{3})+2\pi r_{o}^{2}t_{d}\left(\tau_{y0I}+\eta_{0I}\frac{r_{o}\omega }{h}\right) \end{array}\right\} \end{array}$$

where $\eta_{I}$ is the Ith value of η.

The value of $t_{d}$ in this study was 23 mm, and h ranged from 0 to 2 mm. Note that the transmission torque of the MR friction clutch was obtained by ignoring the torque generated by the friction with the seal and in the bearing. In addition, the electromagnetic time constant for arranging the ferromagnetic particles along the magnetic field lines was 0.4–0.8 ms [36,37]. As such a small delay can be neglected, the response delay to reach the steady-state damping force is typically modeled as a first-order dynamic system [38].

## 3. Design and Magnetic Analysis of Multi-Plate MR Clutch

**Figure 2:**
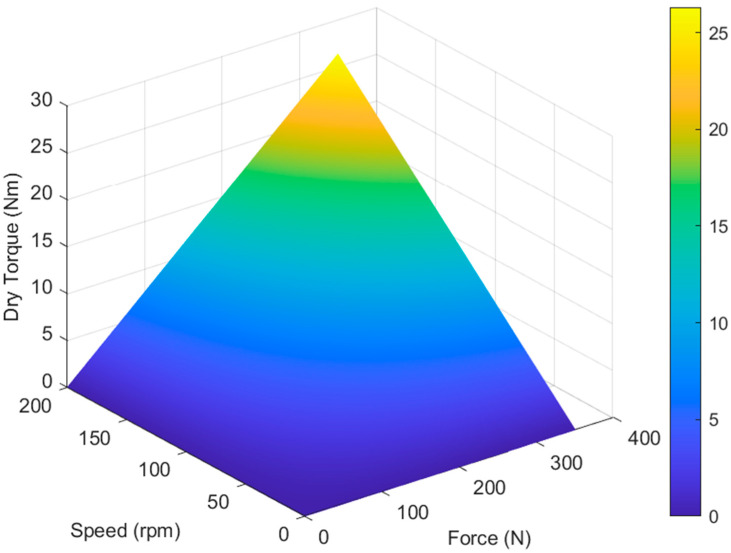
Torque transmission calculation results according to rotational speed and axial compression force.

**Figure 3:**
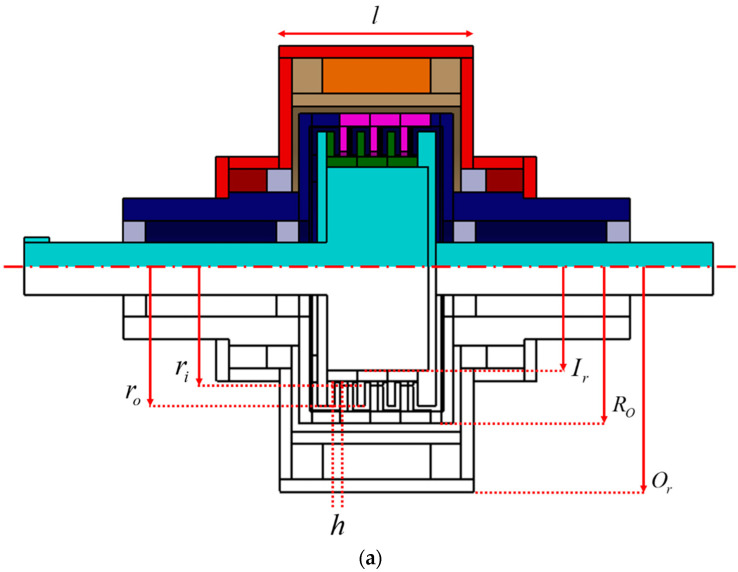

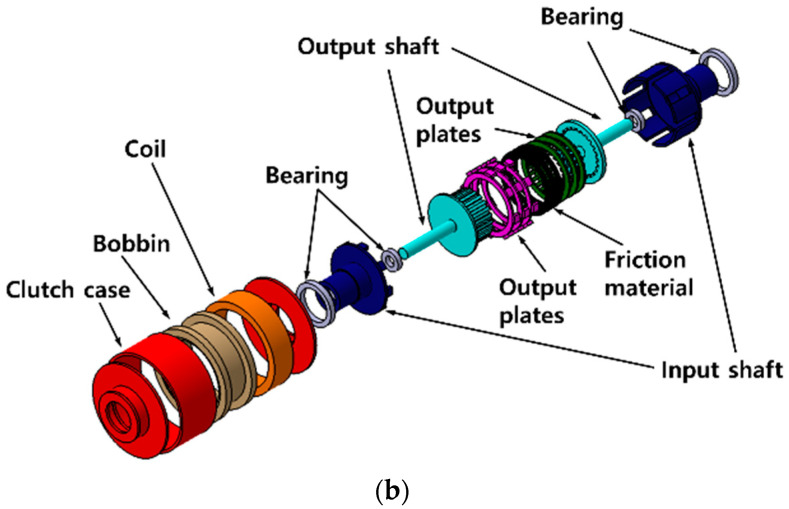
Multi-plate MR clutch (**a**) design parameters and (**b**) model.

The size of the proposed multi-plate MR clutch was designed based on a small-capacity multiplate clutch. To predict the torque transmission of the designed multi-plate MR clutch, the torque generated by the mechanical friction in the clutch was calculated using the torque modeling Equation (8). The axial holding force F was evaluated at 0–350 N and the rotation speed was evaluated at 0–200 rpm, as shown in Figure 2. The objective dry torque was at 0–25 Nm, which is sufficient for power transmission. To meet the objective dry torque requirements, two design variables were established: the motor revolution speed was set to 200 rpm and the compression force was set to 320 N. These values resulted in a calculated dry torque of 26.5 Nm. The MR multi-plate clutch was modeled and fabricated as shown in Figure 3. During the design process, the geometric tolerance was set to 0.1 mm.

The parts of the multi-plate MR clutch were fabricated with a machining tolerance of 0.05 mm, which represents the finest accuracy achievable with common machining tools. During the assembly of the multi-plate MR clutch, a uniform distribution of gaps between the plates was considered for simplicity. The final configuration and dimensions of the designed multi-plate MR clutch are summarized in Figure 3 and Table 2.

Before mathematically evaluating the multi-plate MR clutch torque transmission in magnetic field control mode, an electromagnetic analysis was performed using ANSYS Maxwell to investigate the formation of magnetic fields according to the gap between the disc and plate and the applied current strength. For this analysis, the inner space of the clutch was assumed to be filled by the MR fluid without voids. In addition, the MR fluid mesh was set denser than the clutch mesh to clearly identify the result of the magnetic field acting between the disc and plate, where torque is transmitted. The conditions for this electromagnetic analysis are listed in Table 3.

The finite element model of the multi-plate MR clutch was constructed using the eight-vertice 2D-axisymmetric couple element (PLANE 13) included in the commercial ANSYS software to reflect the magnetic circuits. A total of elements 204,132 were employed in the completed model.

In addition, the temperature increase in the MR fluid arises partially from the fluid friction and partially from the Ohmic losses in the magnetic circuit. The characteristics of the damping force owing to viscous damping reduction at high temperatures were revealed by the ANSYS model. Such changes lead to a model mismatch and degradation of the control effect [39,40]. However, the proposed hybrid multi-plate MR clutch was provided with a torque tracking control mode that is capable of overcoming these changes. Accordingly, degradation owing to heating was neglected in this study.

**Figure 4:**
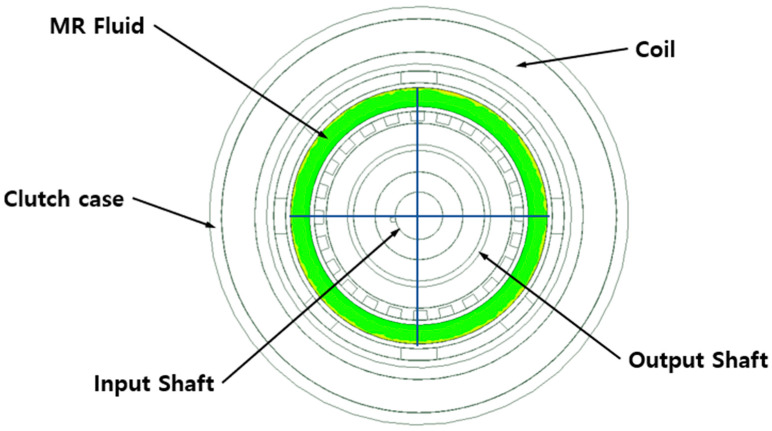
Measurement positions of the average magnetic field density.

**Figure 5:**
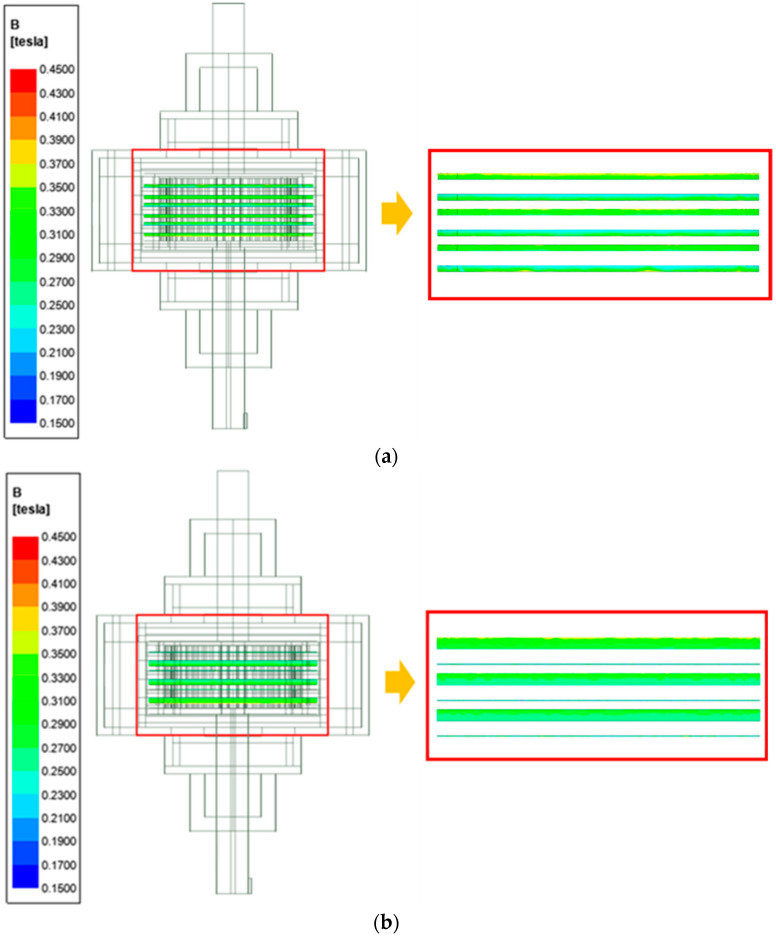
Electromagnetic field analysis results for a gap between the disc and plate of: (**a**) 2.0 and (**b**) 0.4 mm.

To determine the strength of the magnetic field acting on the MR fluid between the clutch plates and discs using the electromagnetic field analysis, the average value in the MR fluid was derived by establishing four measurement locations at the gaps between the disc and plate along perpendicular lines passing through the center of the clutch shafts, as shown in Figure 4. The gap was varied between 0.02 and 2.0 mm in 0.2-mm increments. The results of the analysis are summarized in Figure 5 and Table 4. The magnetic field density reached a maximum of 0.249 T when the gap between the disc and plate was 0.4 mm, and a maximum of 0.297 T when the gap was 2 mm. However, note that when the gap between the disc and plate was 0.4 mm on one side of the disc, a 3.6 mm gap was generated on the opposite side of the disc. Thus, the magnetic field density decreases as the largest distance to the plate on either side of the disc decreases, with an optimum when the gap is equal on both sides of the disc.

**Figure 6:**
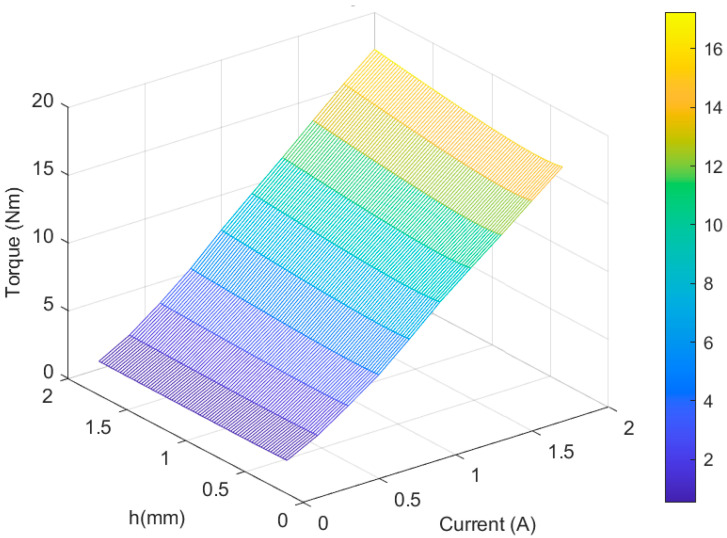
Torque transmission results according to the applied current and gap *h* between disc and plate.

Based on the magnetic field analysis results according to the gap between disc and plate, the *B*–*h* curve, and the shear stress graph of 132 DG MR fluid, when the inner diameters of disc and plate were changed, the magnetic strength governing the transmission torque in magnetic field control mode increased with increasing current. Hence, when the gap was 2 mm, the transmission torque increased from 0.56 to 17.24 Nm as the current increased from 0.2 to 2 A, and when the gap decreased to 0.4 mm, the transmission torque at 2 A decreased from 17.24 to 15.9 Nm. Furthermore, when the gap was 0.62 mm, a small torque of 15.74 Nm was obtained, and when the gap decreased, the transmission torque tended to increase again. This is illustrated in Figure 6.

## 4. Experimental Results and Discussion

### 4.1. Results

**Figure 7:**
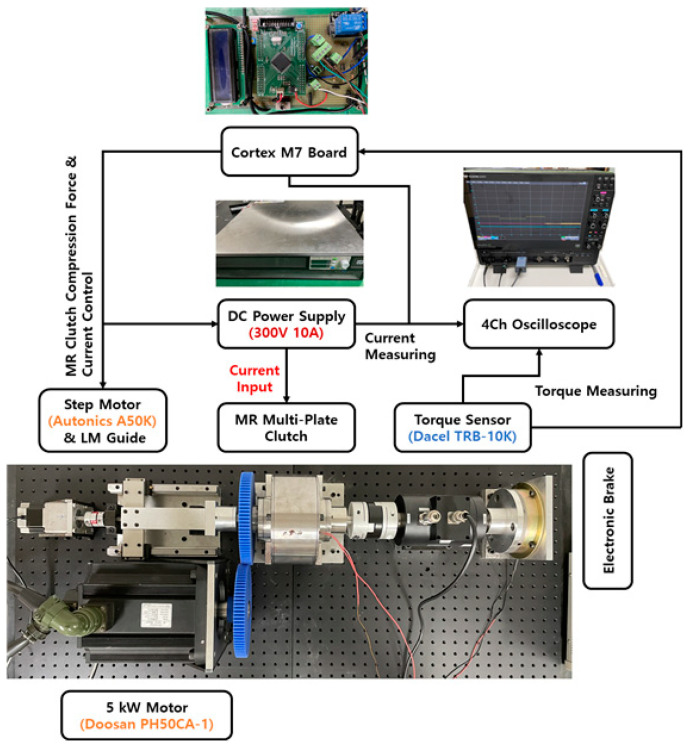
Schematic diagram of the experimental setup.

The multi-plate MR clutch proposed in Section 3 was fabricated and used to conduct torque transmission experiments. As shown in Figure 7, the experimental setup for measuring the transmitted torque comprised a motor to rotate the input shaft and a torque sensor to measure the transmission torque of the output shaft. To manage the control signals and sensor measurements, peripherals were designed to transmit and collect digital and analog signals, and fabricated on a single control board. The Cortex M7 was used as the micro control unit (MCU) for torque control, and the IAR Embedded Workbench was used to run the compiled control program. A motor controller was used for the drive motor control, an electronic brake was used to generate a physical load on the output shaft, and an amplifier was used to amplify the torque sensor signals. In addition, a power supply with 350 V, a 10-A output was used to form a magnetic field in the MR fluid using a coil. To collect the experimental data, the desired torque and applied current were measured using LeCroy’s four-channel oscilloscope WavePro 604 HD (Future Automotive Intelligent Electronics Core Technology Center, Republic of Korea).

Clutch operation could be performed normally at rotational speed as high as 1800 rpm, the maximum speed of the motor; however, in this study, experiments were conducted at a lower rotational speed of 200 rpm for safety. In the clutch experiment, the torque transmission characteristics were examined as the compression force, current, and gap between disc and plate were changed to perform basic evaluations of the magnetic field control and mechanical friction modes of the fabricated multi-plate MR clutch. In magnetic field control mode, the torque transmission was evaluated according to the current and gap between disc and plate. The magnetic field was again applied to the MR fluid by adjusting the current flowing in the coil in the range of 0–2 in 0.2-A increments; the gap was also changed in the range of 0.2–2.0 mm in 0.2-mm increments during the experiment. Finally, the ability of the clutch to transmit a torque of up to 30 Nm through the compression force in mechanical friction mode was investigated.

The transmission torque was evaluated by applying a compression force between the disc and plate while applying a current to the gap using the coil during clutch operation at a constant rpm. The experimental protocols used are listed in Table 5.

**Figure 8:**
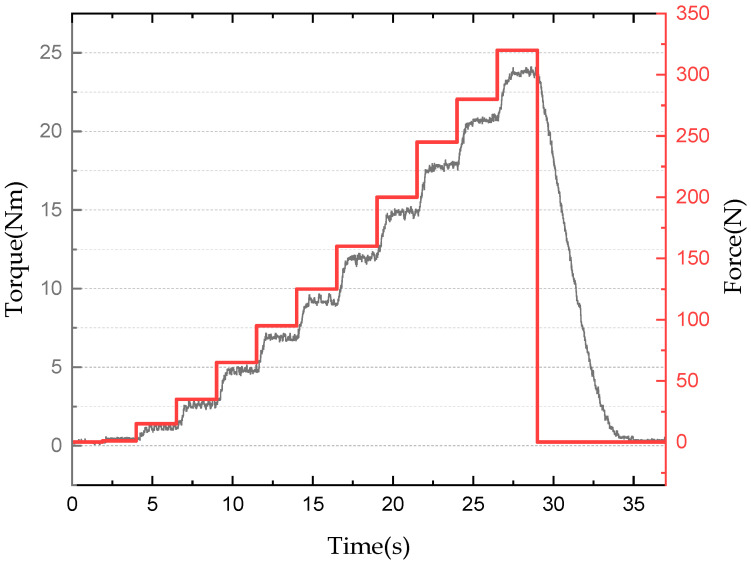
Torque transmission in mechanical friction mode according to applied compression force.

Figure 8 shows the experimental results for proposed multi-plate MR clutch in mechanical friction mode. Measurements of the torque transmitted through the compression force were performed at a motor rotation of 200 rpm until the compression force was removed. Because the compression force could not be measured in mechanical friction mode, it was back calculated by measuring the transmission torque after moving the output shaft 0.2 mm in the axial direction using a step motor and a ball screw at the point of attachment. Detailed results are shown in Table 6. In the mechanical friction mode experiment, the transmission torque increased up to 23.7 Nm starting when compression was applied, exhibiting a maximum steady-state arrival time of 680 ms. Furthermore, a torque ripple of up to 1 Nm was generated, and approximately 5 s were required until the power transmission was blocked from the maximum friction. This indicates that the torque transmission response was delayed owing to the limited rotational torque of the step motor.

**Figure 9:**
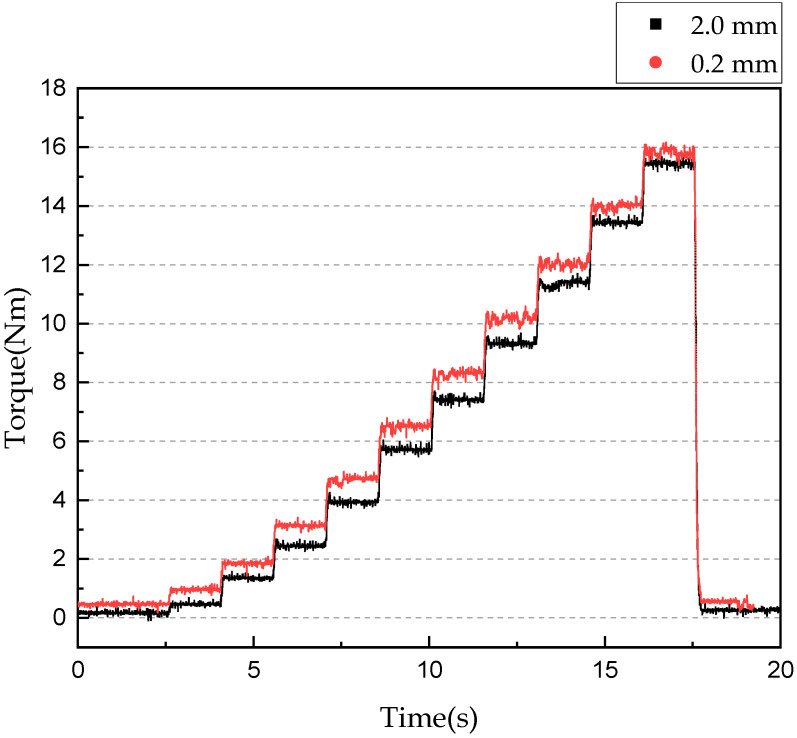
Torque transmission in magnetic field control mode according to applied current.

Figure 9 shows torque transmission results in the magnetic field control mode experiment according to the applied current using the same parameters applied in the mechanical friction mode. The transmission torque was measured as the current was increased from 0 to 2 A in 0.2-A increments at 1.5-s intervals with a gap of 2 or 0.2 mm. The figure shows that the transmission torque changed according to the applied current and provided gap. Detailed results are provided in Table 7. The experimental results indicate that when the gap was fixed at 2 mm and the current was increased from 0.2 to 2 A, the transmission torque increased to a maximum of 15.43 Nm, and the maximum steady-state arrival time was 220 ms. When the gap was 0.2 mm, the maximum transmission torque was 15.93 Nm and the maximum steady-state arrival time was 200 ms. The transmission torque response in magnetic field control mode was notably faster than that in mechanical friction mode. However, a transmission torque ripple of up to 0.76 Nm occurred owing to the vibration resulting from the friction between the disc and plate at a gap of 0.2 mm.

**Figure 10:**
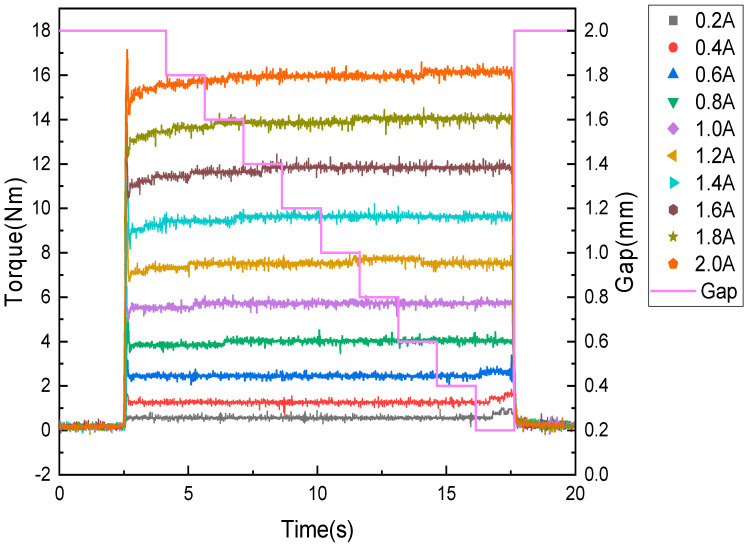
Torque transmission in magnetic field control mode according to changes in gap and current.

Figure 10 shows the transmission torque measured during the magnetic field control mode experiment when the provided gap was decreased from 2.0 to 0.2 mm in 0.2-mm increments at 1.5-s intervals. Similar to Figure 9, Figure 10 shows that the transmission torque changed depending on the current and gap. experimental results describing the change in transmitted torque according to gap and applied current are summarized in Table 8. The transmission torque was 0.7 Nm when the applied current was 2 A and 0.25 Nm when it was 0.2 A. The closer the friction surfaces of the disc and plate, the more the transmission torque tended to increase. At approximately 0.2 mm, immediately before contact, fine friction began to occur and the transmission torque increased further. This friction loss occurred at the oil seal keeping the MR fluid inside the clutch as well as at the thrust bearing used to maintain the gap between the disc and plate and the shaft bearings used to prevent the deflection of the input and output shafts. Thus, it was confirmed that the torque increased as the applied current increased, and increased faster as the gap increased. Furthermore, it was determined that the transmitted torque was affected by friction at low applied currents.

The experiments conducted using the proposed multi-plate MR clutch in mechanical friction mode and magnetic field control mode confirmed that in mechanical friction mode, the torque response time was slow and a torque ripple was generated, whereas in magnetic field control mode, the torque response time was approximately three times faster and the torque ripple was small. Therefore, to quickly and smoothly control the torque tracking of the proposed multi-plate MR clutch, a control strategy should be established that prioritizes magnetic field control and assists torque control using mechanical friction when a large torque is necessary.

### 4.2. Torque Control Strategy

**Figure 11:**
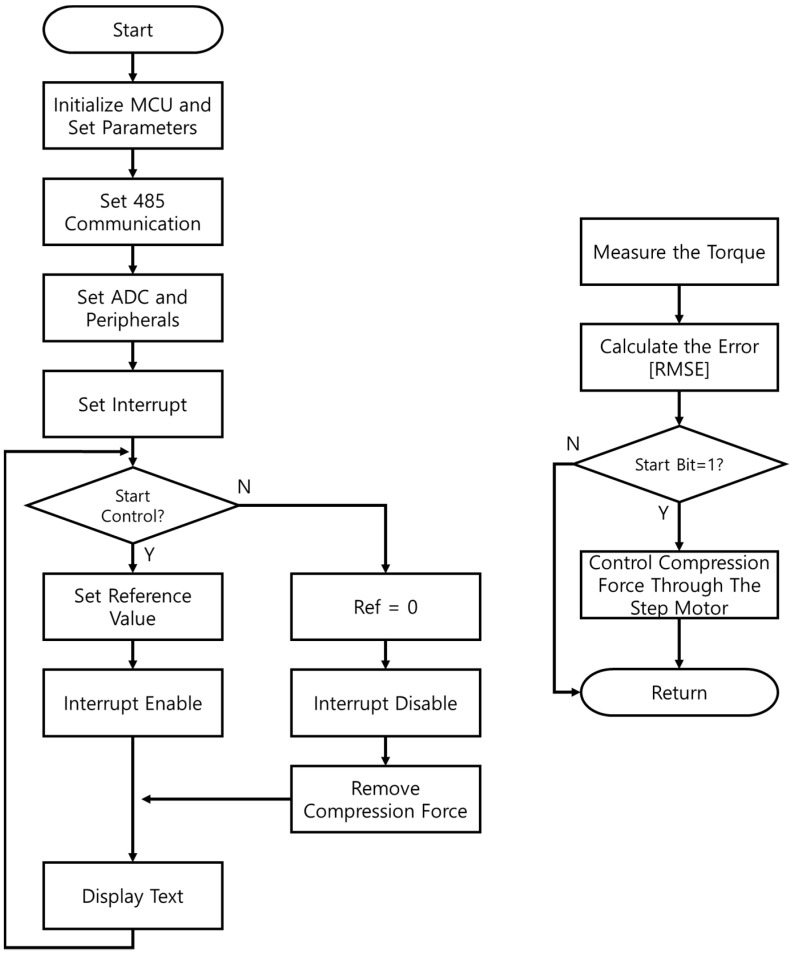
Control algorithm for the multi-plate MR clutch using only mechanical friction mode.

**Figure 12:**
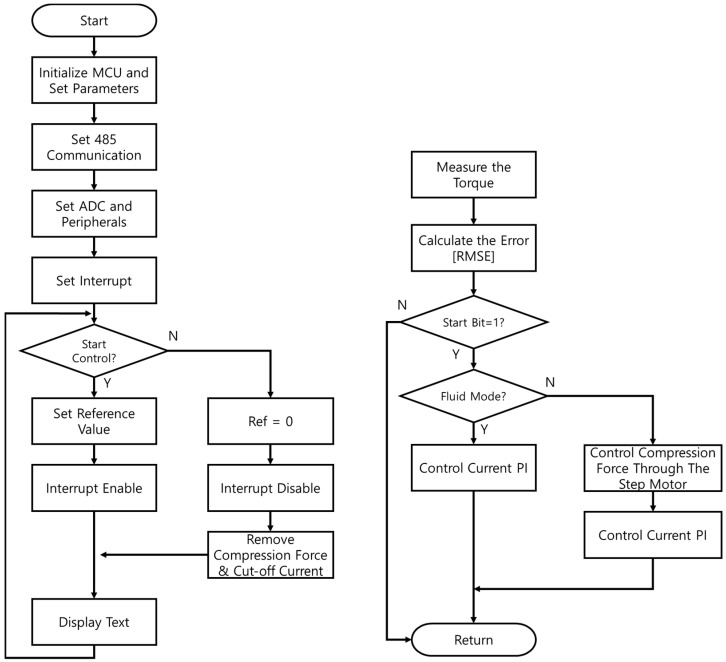
Control algorithm for the multi-plate MR clutch in mixed mechanical friction/magnetic field control mode.

To achieve adequate torque control of the proposed multi-plate MR clutch, torque control performance should be evaluated using the flowcharts for the control algorithms shown in Figure 11 and Figure 12. First, according to the control flowchart when using only the mechanical friction mode (Figure 11), when the program starts, the MCU is initialized, and the parameters are set. Then, a 10-ms interrupt service routine is set with the peripherals. Once all settings are completed, the clutch is in standby mode, and its state is the same as it is under the stop command. When the user gives a start command, tracking control is performed using the desired torque value, and the interrupt service routine is performed every 10 ms. The current torque is then measured, and the error is calculated to control the rotational direction and speed of the step motor.

The initialization stage is the same in the control flowchart for the mixed mode (Figure 12). However, during the interrupt service routine, the control mode of the multi-plate MR clutch can be changed according to the desired torque, and the necessary parameters are separately controlled accordingly.

**Figure 13:**
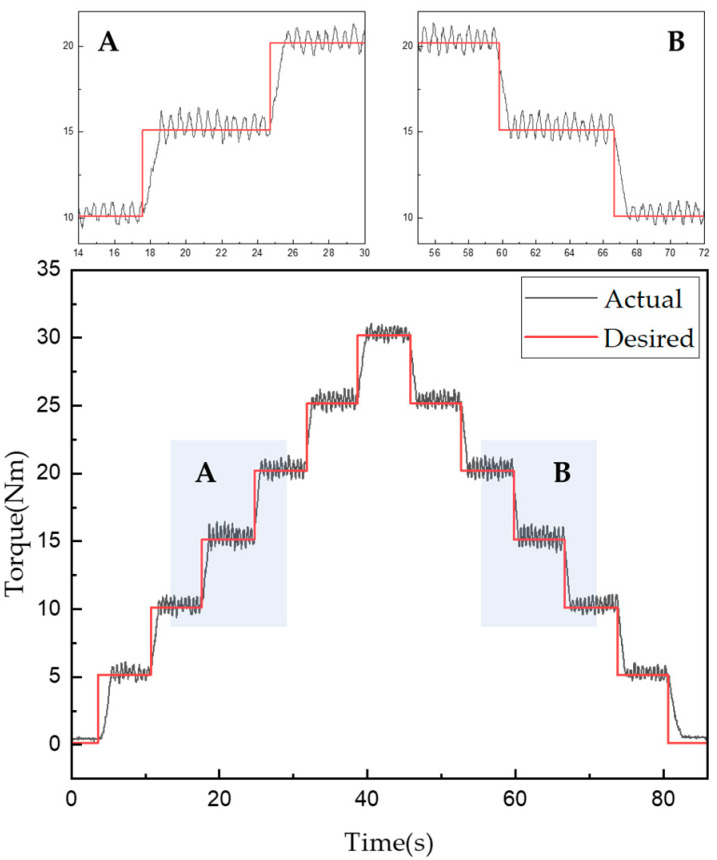
Torque tracking results for step control in mechanical friction mode.

**Figure 14:**
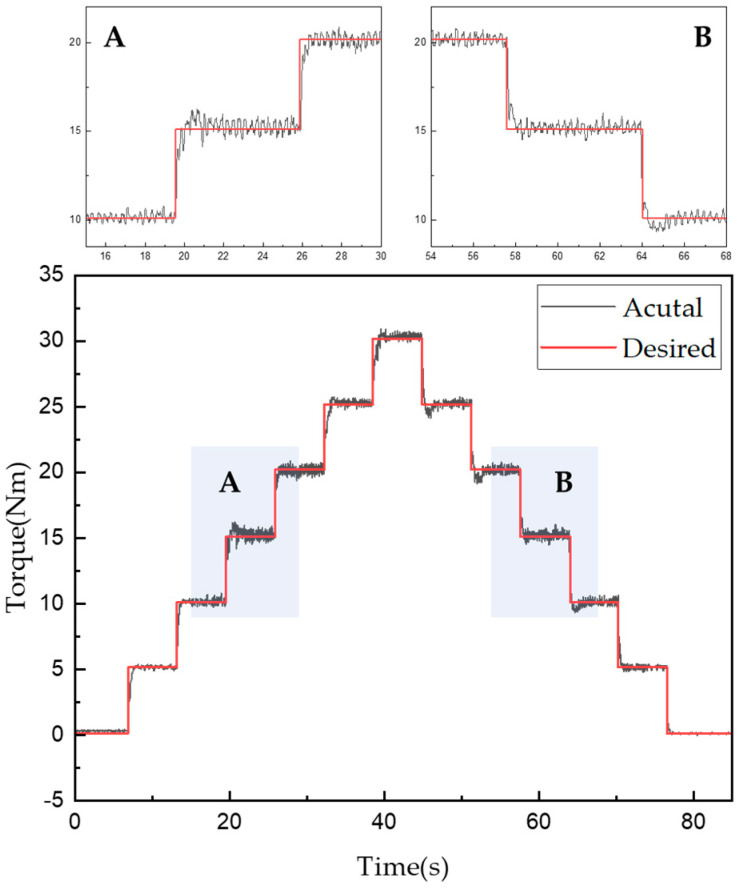
Torque tracking results for step control in mixed mode.

Figure 13 and Figure 14 show the experimentally obtained torque control characteristics when using the control algorithms for mechanical friction mode and mixed mode, respectively. The command was given using the proposed algorithm to increase the torque from 0 to 30 Nm in 5-Nm intervals and then decrease it in the same fashion. Torque tracking using only mechanical friction mode, shown in Figure 13, required a maximum of 1000 ms and minimum of 642 ms after the torque command was issued. In addition, the root mean square error (RMSE) was used to evaluate the control performance as follows:

(13) $$RMSE=\sqrt{\frac{\sum_{i=1}^{n}{\left({\hat{y}}_{i}-y_{i}\right)}^{2}}{n}}$$

where $\hat{y}$ is the measured torque, y is the torque command, and n is the number of data points. Thus, an RMSE of approximately 1.192 Nm was obtained when using mechanical friction mode.

Torque tracking in the mixed mode required a maximum of 870 ms and a minimum 310 ms after the torque command was issued. The RMSE for the torque tracking error was approximately 0.649 Nm. According to the control algorithm in Figure 14, the current control was performed first, then from the moment when the 15 Nm torque command was received, the mixed and mechanical friction modes began to run in parallel. The vibration was found to be more severe when using only the mechanical friction mode than when using the mixed mode. However, the actual torque matched well with the desired torque when using either mode. Furthermore, the control performance in the mixed mode was superior in terms of both the tracking control error and steady-state arrival time. Figure 13 and Figure 14 also show enlarged views of the measured change in torque near the 15-Nm value. Even when the current control mode was switched to the mixed mode, the torque tracking error and vibration for the stepped command values remained very small. The tracking response times for each mode are listed in Table 9 and Table 10.

**Figure 15:**
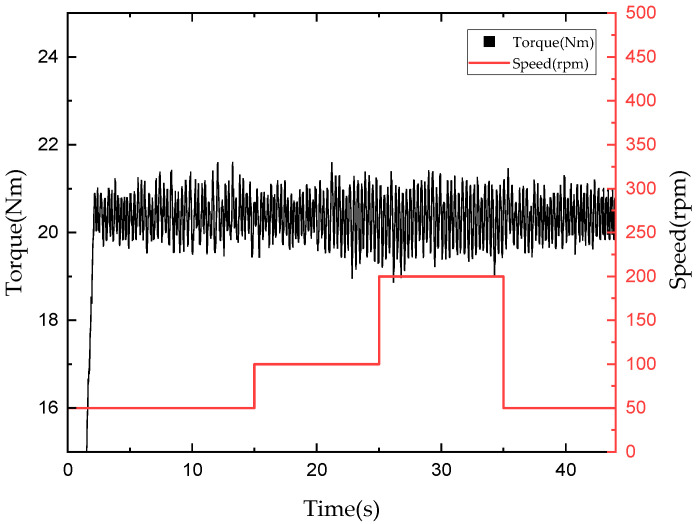
Torque tracking results according to rotational speed in mechanical friction mode.

**Figure 16:**
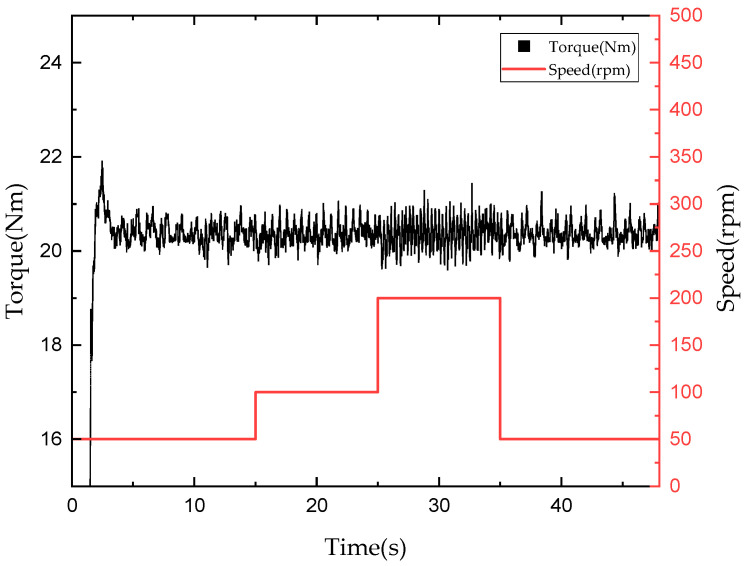
Torque tracking results according to rotational speed in mixed mode.

Figure 15 and Figure 16 depict graphs of the tracking control results for a torque command of 20 Nm when the rotational speed of the input motor was changed from 50 to 100 to 200 to 50 rpm after giving a torque command of 20 Nm. When using mechanical friction mode, a torque ripple was generated by default, and an RMSE for torque tracking of 0.6126 Nm was observed when the rotational speed was changed. Thus, although the torque fluctuated significantly when the rotational speed was changed, torque tracking remained possible. Furthermore, in the mixed mode, an RMSE for torque tracking of approximately 0.437 Nm occurred when the rotational speed was changed. This suggests that, in the mixed mode, the torque can be tracked more smoothly with a smaller error than in the mechanical friction mode, even when the rotational speed is changed.

**Figure 17:**
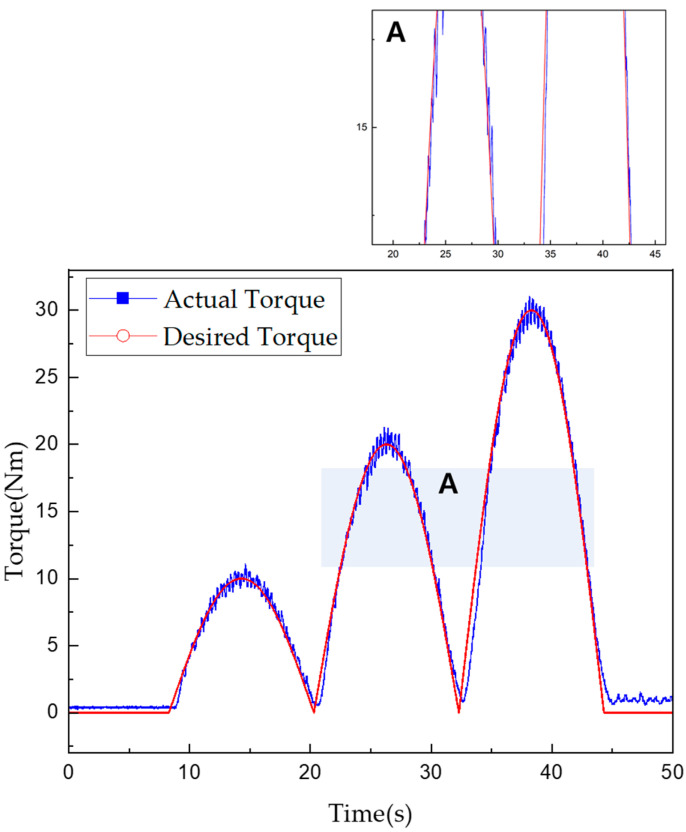
Torque tracking results for half-sine-wave input in mechanical friction mode.

**Figure 18:**
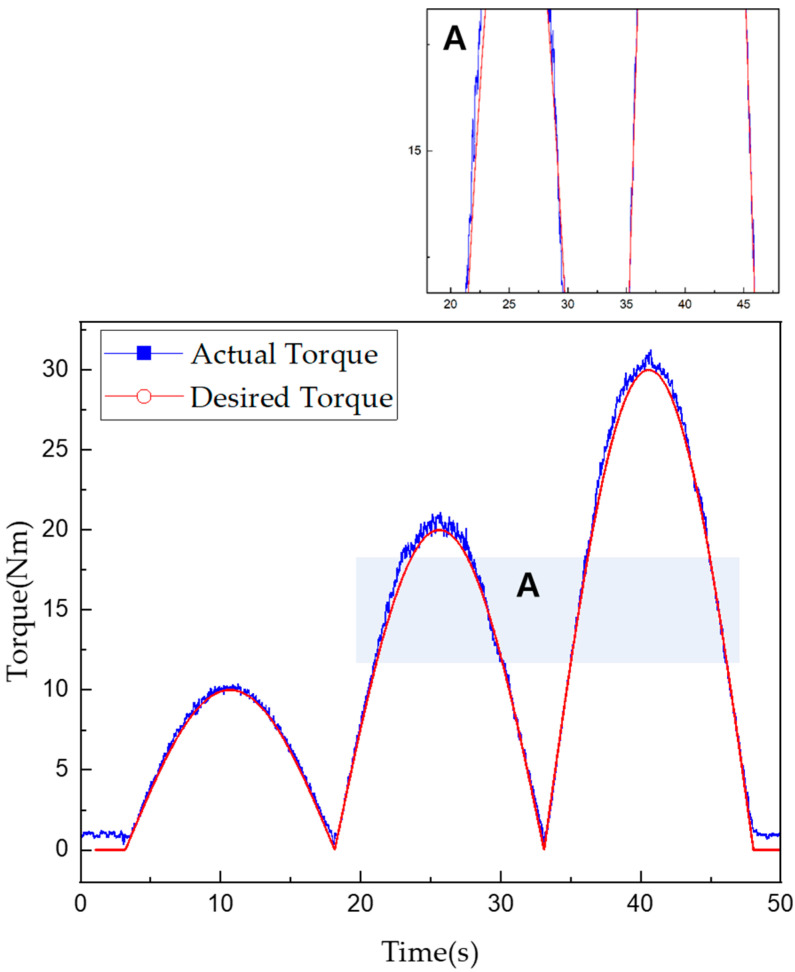
Torque tracking results for half-sine-wave input in mixed mode.

Figure 17 and Figure 18 show torque tracking graphs when half-sine-wave type torque commands of 0–10, 0–20, and 0–30 Nm were provided using the two control modes. When using only the mechanical friction mode, a torque ripple was generated, and the RMSE for torque tracking was 0.866 Nm; however, the possibility of torque tracking for half-sine-wave type torque commands was verified. In the mixed mode, the RMSE for torque tracking was 0.553 Nm. It was assumed that in this case, some noise also existed in the desired torque, and the error of the proportional–integral (PI) controller was small at the beginning when the torque increased, resulting in a tracking delay and tracking error. However, because the torque tracking error remained less than 1.7% at the maximum torque, the torque tracking control was considered to be smoothly conducted.

## 5. Conclusions

This study modeled and evaluated the torque transmission of a multi-plate MR clutch in mechanical friction mode, magnetic field control mode, and a mixed mode. Torque tracking control was then performed using the multi-plate MR clutch based on the derived torque modeling equations and the magnetic field analysis results obtained using ANSYS Maxwell.

The results of the torque performance experiment indicated that the maximum transmitted torque was 24.1 Nm in mechanical friction mode and 15.5 Nm in magnetic field control mode. These values were approximately 2 Nm smaller than their corresponding simulation results of 26 and 17 Nm, respectively. This difference was attributed to the mechanical structure required to create a compression force between the friction surfaces and small errors in the magnetic field analysis. The change in torque according to the applied rotational speed exhibited only a slight change, with a maximum difference of 0.7 Nm observed when compared to the torque model calculation result. The cause of this difference was assumed to be the loss of power owing to friction at the clutch seal and bearings. However, because this loss a small compared to the maximum torque, the torque modeling equation and magnetic field analysis were determined to be reliable.

After establishing a control algorithm and using it to control the torque tracking of the proposed MR multi-plate clutch, the RMSEs in mechanical friction mode for the step command, rotational speed change, and half-sine wave command experiments were 1.192, 0.616, and 0.866 Nm, respectively. Compared with the results in mechanical friction mode, the RMSEs were relatively smaller in the mixed mode. Furthermore, the basic torque ripple was large in mechanical friction mode but smaller in mixed mode. Indeed, in mixed mode, a relatively large RMSE value appeared in the half-sine-wave experiment, but the tracking error was small, except for an initial increase. This indicates that the proposed MR multi-plate clutch can be applied to realize large and accurate power transmission systems.

The proposed multi-plate MR clutch was shown to operate smoothly by capitalizing on the advantages of both dry and wet clutches. In other words, the proposed multi-plate MR clutch reduced vibration by performing smooth shifting operations without torque ripples compared to a purely mechanical friction clutch and exhibited excellent power transmission performance similar to that of a conventional multiplate clutch. These properties are expected to be advantageous in a variety of fields.

In future research, the maximum performance of the clutch could be realized if the drive motor and the step motor controlling the compression force are replaced with equivalents having a higher capacity and rotation speed while producing less noise.

## Figures and Tables

**Table 1:** Rheological properties of the MRF-132DG MR fluid.

Parameter	Value
Natural state of viscosity coefficient, $\eta_{0}$	0.1 [pa·s]
Saturated state of viscosity coefficient, $\eta_{\infty }$	3.8 [pa·s]
Saturated moment for viscosity coefficient, $\sigma_{S\eta }$	4.5 [T^−1^]
Natural state of yield stress, $\tau_{y0}$	12 [pa]
Saturated state of yield stress, $\tau_{y\infty }$	40,000 [pa]
Saturated moment for yield stress, $\sigma_{S\tau_{y}}$	2.9 [T^−1^]
Density	2.95–3.15 [g/cm^3^]
Solids content by weight	80.98%
Operating temperature	−40 to 130 [°C]

**Table 2:** Geometry of the proposed hybrid multi-plate MR clutch.

Parameter	Description	Value [mm]
$O_{r}$	Radius of output shaft	61
$I_{r}$	Radius of input shaft	44
$r_{i}$	Inner radius of clutch plate and disc	45
$r_{o}$	Outer radius of clutch plate and disc	55
h	Disc-to-plate gap	2
$R_{o}$	Outer radius of multi-plate MR clutch	88
ℓ	Length of multi-plate MR clutch except shaft	77

**Table 3:** Multi-plate MR clutch electromagnetic field analysis conditions.

Parameter	Value
Number of turns	1000
Coil resistance	22 [Ω]
Current	0–2 [A]
Mesh	200,000

**Table 4:** Average magnetic field density according to the gap between disc and plate.

Gap [mm]	Applied Current [A]									
	0.2	0.4	0.6	0.8	1.0	1.2	1.4	1.6	1.8	2.0
	Magnetic field density [T]									
2.0	0.035	0.067	0.099	0.129	0.161	0.189	0.216	0.244	0.271	0.297
0.4	0.028	0.056	0.082	0.106	0.133	0.159	0.182	0.204	0.227	0.249

**Table 5:** Experimental protocols and simulation parameters.

Title of Experiment	Input Condition	Current Input
Mechanical friction mode	Compression force 15–320 [N]	0 [A] (fixed)
Magnetic field control mode	Gap 0.2–2 [mm]	0–2 [A]
Mechanical friction mode for torque tracking control	Determined by control algorithm	0 [A] (fixed)
Mixed mode for torque tracking control	Determined by control algorithm	

**Table 6:** Torque transmission experiment results in mechanical friction mode according to applied compression force.

Applied Force [N]	15	35	65	95	125	160	200	245	280	320
Torque [Nm]	1.03	2.43	4.75	6.94	9.2	11.8	14.75	18.01	20.77	23.7
Response time [ms]	639	545	667	657	569	655	680	578	602	576
Torque ripple [Nm]	0.63	0.81	0.7	0.77	0.84	0.92	0.94	0.73	1.03	0.78

**Table 7:** Torque transmission experiment results in magnetic field control mode according to applied current with 2.0- and 0.2-mm gaps.

Gap between Disc and Plate	Applied Current [A]									
	0.2	0.4	0.6	0.8	1.0	1.2	1.4	1.6	1.8	2.0
	Transmitted torque [Nm]									
2.0 [mm]	0.41	1.28	2.42	3.95	5.75	7.48	9.44	11.4	13.5	15.4
0.2 [mm]	1.05	1.93	3.17	4.84	6.59	8.25	10.2	12.1	14.1	15.9
	Response time [ms]									
2.0 [mm]	146	137	205	195	162	179	203	220	195	152
0.2 [mm]	148	134	132	134	146	183	186	213	177	200
	Torque ripple [Nm]									
2.0 [mm]	0.43	0.34	0.39	0.51	0.54	0.55	0.55	0.60	0.47	0.51
0.2 [mm]	0.38	0.64	0.50	0.56	0.66	0.76	0.76	0.64	0.66	0.76

**Table 8:** Torque transmission experiment results in magnetic field control mode according to current at gaps of 2 and 0.2 mm.

Gap between Disc and Plate	Applied Current [A]									
	0.2	0.4	0.6	0.8	1.0	1.2	1.4	1.6	1.8	2.0
	Transmitted torque [Nm]									
2.0 [mm]	0.64	1.3	2.53	3.88	5.58	7.3	9.4	11.4	13.4	15.6
0.2 [mm]	0.89	1.47	2.78	4.2	5.85	7.67	9.8	11.8	14	16.3

**Table 9:** Torque tracking response times according to step control in mechanical friction mode.

Desired Torque [Nm]	5	10	15	20	25	30	25	20	15	10	5	0
Response time [ms]	1000	740	680	710	660	874	738	769	722	642	781	854

**Table 10:** Torque tracking response times according to step control in mixed mode.

Desired Torque [Nm]	5	10	15	20	25	30	25	20	15	10	5	0
Response time [ms]	750	395	538	456	870	780	551	563	601	532	522	310

## References

[1] Vasca F., Iannelli L., Senatore A., Scafati M.T. Modeling torque transmissibility for automotive dry clutch engagement. Proceedings of the 2008 American Control Conference.

[2] Jibin H., Zengxiong P., Chao W. (2012). Experimental research on drag torque for single-plate wet clutch. J. Tribol..

[3] D’Agostino V., Cappetti N., Pisaturo M., Senatore A. (2012). Improving the engagement smoothness through multi-variable frictional map in automated dry clutch control. ASME International Mechanical Engineering Congress and Exposition.

[4] Desmidt H., Smith E.C., Bill R.C., Rao S. Multi-Plate Dry Clutch Design and Analysis for Dual Speed Rotorcraft Applications. Proceedings of the 56th AIAA/ASCE/AHS/ASC Structures, Structural Dynamics, and Materials Conference.

[5] Arief I., Mukhopadhyay P.K. (2019). Magnetorheology in CoNi nanoplatelet-based MRFs: Effect of platelet orientation and oscillatory shear. J. Magn. Magn. Mater..

[6] Neelakantan V.A., Washington G.N. (2005). Modeling and reduction of centrifuging in magnetorheological (MR) transmission clutches for automotive applications. J. Intell. Mater. Syst. Struct..

[7] Benetti M., Dragoni E. (2006). Nonlinear magnetic analysis of multi-plate magnetorheological brakes and clutches. In Proceedings of the COMSOL Users Conference.

[8] Gratzer F., Steinwender H., Kušej A. (2008). Magnetorheological AWD clutches. ATZautotechnology.

[9] Kikuchi T., Ikeda K., Otsuki K., Kakehashi T., Furusho J. (2009). Compact MR fluid clutch device for human-friendly actuator. J. Phys. Conf. Ser..

[10] Saifei Z., Yong L. Disc Shaped High-Torque-MRF-Clutch Design. Proceedings of the International Conference on Computer Application and System Modeling.

[11] Güth D., Aust M., Maas J. Novel concepts for MRF based clutch systems with integrative functionalities. Proceedings of the 2010 IEEE/ASME International Conference on Advanced Intelligent Mechatronics.

[12] Törőcsik D. (2011). Some design issues of multi-plate magnetorheological clutches. Hung. J. Ind. Chem..

[13] Wang D., Tian Z., Meng Q., Hou Y. (2013). Development of a novel two-layer multiplate magnetorheological clutch for high-power applications. Smart Mater. Struct..

[14] Kavlicoglu B.M., Gordaninejad F., Evrensel C.A., Cobanoglu N., Liu Y., Fuchs A., Korol G. (2002). High-torque magnetorheological fluid clutch. Smart Structures and Materials 2002: Damping and Isolation.

[15] Kavlicoglu B.M., Gordaninejad F., Wang X. (2013). Study of a magnetorheological grease clutch. Smart Mater. Struct..

[16] Thakur M.K., Sarkar C. Development and performance analysis of a Magnetorheological fluid Clutch. Proceedings of the COMSOL Conference.

[17] Yadmellat P., Kermani M.R. (2013). Adaptive modeling of a magnetorheological clutch. IEEE/ASME Trans. Mechatron..

[18] Bucchi F., Forte P., Frendo F. (2015). Temperature effect on the torque characteristic of a magnetorheological clutch. Mech. Adv. Mater. Struct..

[19] Pilch Z. (2015). Analysis of Established Thermal Conditions for Magnetorheological Clutch for Different Loading Conditions. Analysis and Simulation of Electrical and Computer Systems.

[20] Leong F.H., Tsai N.C., Chiu H.L. (2017). Infinite-stage magnetic clutch for shaft speed amplification. Proc. Inst. Mech. Eng. Part C J. Mech. Eng. Sci..

[21] Fernando N. A Field Modulated Electromagnetic Clutch with Slip Control. Proceedings of the 2016 IEEE 2nd Annual Southern Power Electronics Conference (SPEC).

[22] Kavlicoglu B., Gordaninejad F., Evrensel C., Fuchs A., Korol G. (2006). A semi-active, high-torque, magnetorheological fluid limited slip differential clutch. J. Vib. Acoust..

[23] Rizzo R., Musolino A., Bucchi F., Forte P., Frendo F. (2015). A multi-gap magnetorheological clutch with permanent magnet. Smart Mater. Struct..

[24] Latha K.H., Sri P.U., Seetharamaiah N. (2017). Design and manufacturing aspects of magneto-rheological fluid (MRF) clutch. Mater. Today Proc..

[25] Olszak A., Osowski K., Kesy Z., Kesy A. (2019). Investigation of hydrodynamic clutch with a magnetorheological fluid. J. Intell. Mater. Syst. Struct..

[26] Zhang H., Du H., Sun S., Li W., Wang Y. (2019). Design and Analysis of a Novel Magnetorheological Fluid Dual Clutch for Electric Vehicle Transmission (No. 2019-01-5014). SAE Tech. Paper.

[27] Kluszczyński K., Pilch Z. Basic features of MR clutches-resulting from different number of discs. Proceedings of the 2019 15th Selected Issues of Electrical Engineering and Electronics (WZEE).

[28] Park J.Y., Kim G.W., Oh J.S., Kim Y.C. (2021). Hybrid multi-plate magnetorheological clutch featuring two operating modes: Fluid coupling and mechanical friction. J. Intell. Mater. Syst. Struct..

[29] Oh J.S., Choi S.H., Choi S.B. (2014). Design of a 4-DOF MR haptic master for application to robot surgery: Virtual environment work. Smart Mater. Struct..

[30] Han Y.M., Oh J.S., Kim S., Choi S.B. (2017). Design of multi-degree motion haptic mechanisms using smart fluid-based devices. Mech. Based Des. Struct. Mach..

[31] Nguyen Q.H., Choi S.B. (2012). Optimal design of a novel hybrid MR brake for motorcycles considering axial and radial magnetic flux. Smart Mater. Struct..

[32] Quoc N.V., Tuan L.D., Hiep L.D., Quoc H.N., Choi S.B. (2019). Material characterization of MR fluid on performance of MRF based brake. Front. Mater..

[33] Vékás L. (2008). Ferrofluids and magnetorheological fluids. Advances in Science and Technology.

[34] https://www.lord.com/sites/default/files/Documents/TechnicalDataSheet/DS7015_MRF-132DGMRFluid.pdf.

[35] Nguyen P.B., Choi S.B. (2013). Accurate torque control of a bi-directional magneto-rheological actuator considering hysteresis and friction effects. Smart Mater. Struct..

[36] Yoon D.S., Kim G.W., Choi S.B. (2021). Response time of magnetorheological dampers to current inputs in a semi-active suspension system: Modeling, control and sensitivity analysis. Mech. Syst. Signal Process..

[37] Yoon D.S., Park Y.J., Choi S.B. (2019). An eddy current effect on the response time of a magnetorheological damper: Analysis and experimental validation. Mech. Syst. Signal Process..

[38] Yang G., Spencer B.F., Carlson J.D., Sain M.K. (2002). Large-scale MR fluid dampers: Modeling and dynamic performance considerations. Eng. Struct..

[39] Priya C.B., Gopalakrishnan N. (2019). Temperature dependent modelling of magnetorheological (MR) dampers using support vector regression. Smart Mater. Struct..

[40] Sahin I., Cesmeci S., Wereley N.M. (2011). Sensitivity of magnetorheological damper behavior to perturbations in temperature via Bouc-Wen model. Electro-Rheological Fluids and Magneto-Rheological Suspensions.

